# Host Range of Influenza A Virus H1 to H16 in Eurasian Ducks Based on Tissue and Receptor Binding Studies

**DOI:** 10.1128/JVI.01873-20

**Published:** 2021-02-24

**Authors:** Josanne H. Verhagen, Per Eriksson, Lonneke Leijten, Ola Blixt, Björn Olsen, Jonas Waldenström, Patrik Ellström, Thijs Kuiken

**Affiliations:** a Department of Biology and Environmental Science, Centre for Ecology and Evolution in Microbial Model Systems (EEMiS), Linnaeus University, Kalmar, Sweden; b Department of Viroscience, Erasmus Medical Center, Rotterdam, the Netherlands; c Zoonosis Science Center, Department of Medical Biochemistry and Microbiology (IMBIM), Uppsala University, Uppsala, Sweden; d Copenhagen Center for Glycomics, Department of Cellular and Molecular Medicine, University of Copenhagen, Copenhagen, Denmark; e Zoonosis Science Center, Department of Medical Sciences, Uppsala University, Uppsala, Sweden; St. Jude Children's Research Hospital

**Keywords:** avian influenza, wild birds, chicken, virus attachment, hemagglutinin, sialic acid receptor, glycan, avian influenza

## Abstract

Dabbling and diving ducks partly occupy shared habitats but have been reported to play different roles in wildlife infectious disease dynamics. Influenza A virus (IAV) epidemiology in wild birds has been based primarily on surveillance programs focused on dabbling duck species, particularly mallard (Anas platyrhynchos). Surveillance in Eurasia has shown that in mallards, some subtypes are commonly (H1 to H7 and H10), intermediately (H8, H9, H11, and H12), or rarely (H13 to H16) detected, contributing to discussions on virus host range and reservoir competence. An alternative to surveillance in determining IAV host range is to study virus attachment as a determinant for infection. Here, we investigated the attachment patterns of all avian IAV subtypes (H1 to H16) to the respiratory and intestinal tracts of four dabbling duck species (*Mareca* and *Anas* spp.), two diving duck species (*Aythya* spp.), and chicken, as well as to a panel of 65 synthetic glycan structures. We found that IAV subtypes generally showed abundant attachment to colon of the *Anas* duck species, mallard, and Eurasian teal (Anas crecca), supporting the fecal-oral transmission route in these species. The reported glycan attachment profile did not explain the virus attachment patterns to tissues but showed significant attachment of duck-originated viruses to fucosylated glycan structures and H7 virus tropism for Neu5Gc-LN. Our results suggest that *Anas* ducks play an important role in the ecology and epidemiology of IAV. Further knowledge on virus tissue attachment, receptor distribution, and receptor binding specificity is necessary to understand the mechanisms underlying host range and epidemiology of IAV.

**IMPORTANCE** Influenza A viruses (IAVs) circulate in wild birds worldwide. From wild birds, the viruses can cause outbreaks in poultry and sporadically and indirectly infect humans. A high IAV diversity has been found in mallards (Anas platyrhynchos), which are most often sampled as part of surveillance programs; meanwhile, little is known about the role of other duck species in IAV ecology and epidemiology. In this study, we investigated the attachment of all avian IAV hemagglutinin (HA) subtypes (H1 to H16) to tissues of six different duck species and chicken as an indicator of virus host range. We demonstrated that the observed virus attachment patterns partially explained reported field prevalence. This study demonstrates that dabbling ducks of the *Anas* genus are potential hosts for most IAV subtypes, including those infecting poultry. This knowledge is useful to target the sampling of wild birds in nature and to further study the interaction between IAVs and birds.

## Abstract (precis)

Influenza A viruses (IAVs) circulate in wild birds worldwide. From wild birds, the viruses can cause outbreaks in poultry and sporadically and indirectly infect humans.

## INTRODUCTION

Host range is a key determinant for the dispersal of infectious agents and plays an important role in their epidemiology and evolution (1). The epidemiology of influenza A virus (IAV) is characterized by a broad host range, including humans, swine, horses, marine mammals, and birds (2). Influenza A viruses are categorized into subtypes based on their surface proteins hemagglutinin (HA; H1 to H18) and neuraminidase (NA; N1 to N11) (2, 3). The largest IAV subtype diversity is seen in wild birds, in which 16 different HA subtypes (H1 to H16) and 9 different NA subtypes (N1 to N9) have been identified (2, 4). Wild bird surveillance activities have shown that wild waterfowl of the order Anseriformes (mainly ducks, geese, and swans) and gulls and shorebirds in the order Charadriiformes are the main reservoirs of IAVs (5–9). Historically, nearly all IAVs in wild waterfowl have had low pathogenicity for chickens and are therefore termed low pathogenic avian influenza A viruses (LPAIVs) (2). LPAIV of the H5 and H7 subtypes can evolve into highly pathogenic avian influenza viruses (HPAIVs) upon introduction into poultry, causing up to 100% mortality in poultry species. The host range of IAVs is partially determined by the HA subtype, as most clearly demonstrated for H13 and H16 subtypes, which have evolved into gull-adapted lineages (9–11) and resulted in host range restriction between species belonging to the Laridae family and Anseriformes order. Similarly, LPAIVs have evolved into Eurasian and American genetic lineages due to long-term geographical separation of host species populations.

For the epidemiology of most IAV subtypes, a major role is played by ducks—in particular, dabbling ducks such as mallard (Anas platyrhynchos)—in which epidemics of LPAIVs occur each fall in the Northern Hemisphere (2, 6). In mallards, LPAIVs replicate in the epithelial cells lining the intestinal tract (12). The viruses are excreted in feces and transmitted to new hosts via the fecal-oral route (12, 13). In mallards, LPAIV prevalence differs strongly by subtype (14), for which an explanation is lacking. Based on 20 years of surveillance of mallards in Eurasia, we considered the subtypes detected in at least 15 of 20 years of surveillance (≥75%) to be “common” (i.e., H1 to H7 and H10) and subtypes detected in less than 5 of 20 years of surveillance (<25%) to be “rare” (i.e., H13 to H16). Subtypes detected in a minimum of 5 and a maximum of 14 of 20 years of surveillance (25% to 75%) were considered “intermediate” subtypes (i.e., H8, H9, H11, and H12) (Tables 1 and 2). This leads to the question of whether mallards are the primary reservoir of the so-called intermediate and rare subtypes or if persistence of these subtypes is driven by infections in other host species, in particular other dabbling (e.g., *Anas* and *Mareca*) or diving (e.g., *Aythya*) duck species, which are insufficiently covered in surveillance studies (15). For instance, in addition to mallards, LPAIVs of diverse HA subtypes have been isolated occasionally to rarely from dabbling ducks such as Eurasian teal (Anas crecca), Eurasian wigeon (Mareca penelope), and gadwall (Mareca strepera) and from diving ducks such as common pochard (Aythya ferina) and tufted duck (Aythya fuligula) (Table 2). The detection of a range of LPAIV subtypes from different nonmallard duck species suggests involvement in IAV epidemiology, but their exact role is poorly understood.

**TABLE 1:** Distribution of low pathogenic avian influenza virus subtypes H1 to H16 in mallards (Anas plathyrynchos) sampled in Eurasia from 1999 to 2018^*a*^

Yr	No. of virus isolates per HA subtype																Total no. of virus isolates
	H1	H2	H3	H4	H5	H6	H7	H8	H9	H10	H11	H12	H13	H14	H15	H16	
1999	3	7	1	1	2	1	1	—	—	—	3	—	—	—	—	—	19
2000	—	—	1	—	1	2	1	—	—	1	—	—	—	—	—	—	6
2001	—	2	4	3	—	2	5	—	1	—	—	—	—	—	—	—	17
2002	7	13	2	18	24	2	33	2	—	17	7	2	—	—	—	—	127
2003	1	11	7	16	5	7	23	5	—	4	2	3	—	—	—	—	84
2004	—	3	5	5	2	7	7	4	2	6	2	—	—	—	—	—	43
2005	8	18	14	32	5	15	12	3	5	10	7	3	—	—	—	—	132
2006	25	4	10	69	22	13	3	11	2	18	5	3	1	—	—	—	186
2007	7	7	17	34	14	17	9	5	17	12	10	3	—	—	—	—	152
2008	3	4	35	69	11	15	17	1	9	14	23	3	—	—	—	—	204
2009	17	5	5	89	15	46	12	3	10	31	38	3	—	—	—	—	274
2010	7	4	43	9	2	6	15	—	4	16	2	—	1	—	1	—	110
2011	4	4	15	18	2	11	17	—	6	9	2	2	—	—	—	—	90
2012	10	1	10	6		5	15	—	1	5	2	—	—	—	6	—	61
2013	4	1	22	8	1	10	20	—	—	4	—	—	1	—	—	1	72
2014	17	2	12	10	3	11	2	—	—	8	7	—	—	—	—	—	72
2015	8	2	28	12	2	3	15	1	—	13	1	—	—	—	—	—	85
2016	—	1	2	21	1	3	2	—	2	1	—	—	—	—	—	—	33
2017	—	—	4	6	—	—	—	—	6	—	—	—	—	—	—	—	16
2018	1	—	—	—	1	—	2	—	—	—	—	—	—	—	—	—	4
																	
Total no. of virus isolates	122	89	237	426	113	176	211	35	65	169	111	22	3	0	7	1	1,787
No. (%) of yrs in which HA subtype was detected	15 (75)	17 (85)	19 (95)	18 (90)	16 (80)	18 (90)	19 (95)	9 (45)	12 (60)	16 (80)	14 (70)	8 (40)	3 (15)	0 (0)	2 (10)	1 (5)	

a Distribution is based on strains in the Influenza Research Database (https://www.fludb.org/; accessed 14 January 2020). —, HA subtype not reported.

**TABLE 2:**
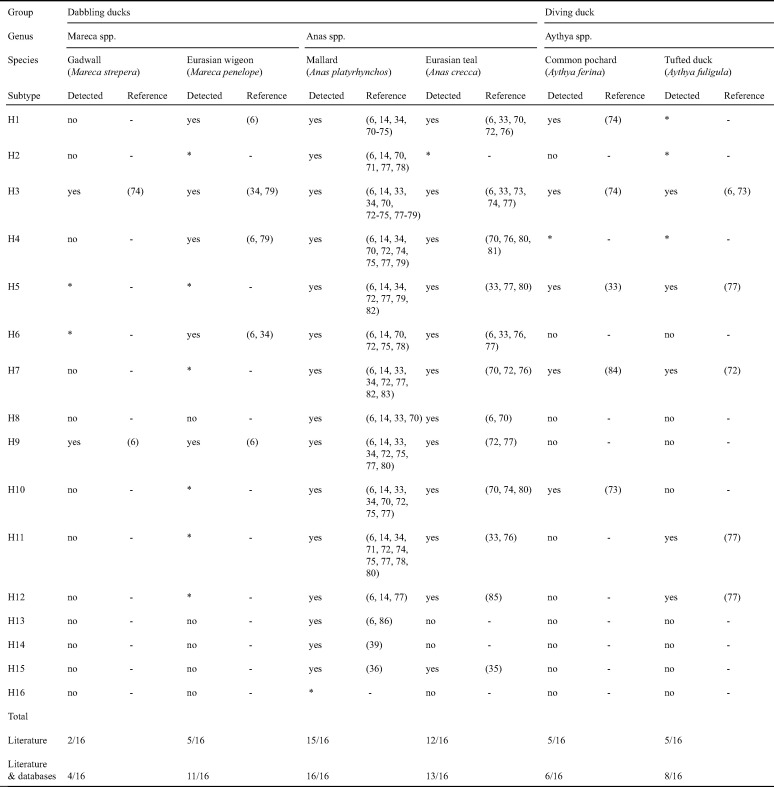
Detection of low pathogenic avian influenza virus subtypes H1 to H16 in six duck species sampled in Eurasia^*a*^

a This table is based on large-scale surveillance studies with no overlapping data sets in time or space and available sequences in online databases. See references 6, 14, 33 to 36, 39, and 70 to 86 as cited in this table. An asterisk indicates that only the sequence of the respective HA subtype was available through the Influenza Research Database (https://www.fludb.org/; accessed 9 September 2019) and/or GenBank (https://www.ncbi.nlm.nih.gov/genbank/; accessed 9 September 2019). –, subtype not detected in literature.

In addition to host range based on LPAIV prevalence data, host range can be assessed based on the host specificity of the HA protein that binds to cells facilitating subsequent cell entry (2). Therefore, the interaction of the HA protein with receptors on the host cell surface is a critical determinant of infection. The HA protein interacts with sialic acid (SA), in particular *N*-acetylneuraminic acid (Neu5Ac), which can be displayed at the termini of free, secreted, membrane-bound, and intracellular glycans (16). The SA is linked to galactose (Gal) via either an α2–3 or α2–6 glycosidic bond. Avian IAVs attach both to α2–3-linked SA and α2–6-linked SA, while human IAVs prefer α2–6-linked SA (17–20). Attachment patterns of IAVs to different glycan structures can be investigated by the use of glycan arrays (21). The suggested main glycan receptors of LPAIVs are 3′STF, 3′SLN, sulfated structure analogues (i.e., Su-3′SLN), and sialylated Lewis structures (i.e., SLe^c^, Su-SLe^c^) (Table 3) (20, 22–26). Moreover, attachment patterns of some HA subtypes (i.e., H3, H4, H6, H12, and H16) of LPAIVs to cells and tissues of birds have been described using virus histochemistry and have been suggested to vary among different HA subtypes and hosts (Table 4) (27–30). Yet, a comparative study on IAV attachment patterns to tissues and receptor structures including all avian HA subtypes is lacking.

**TABLE 3:**
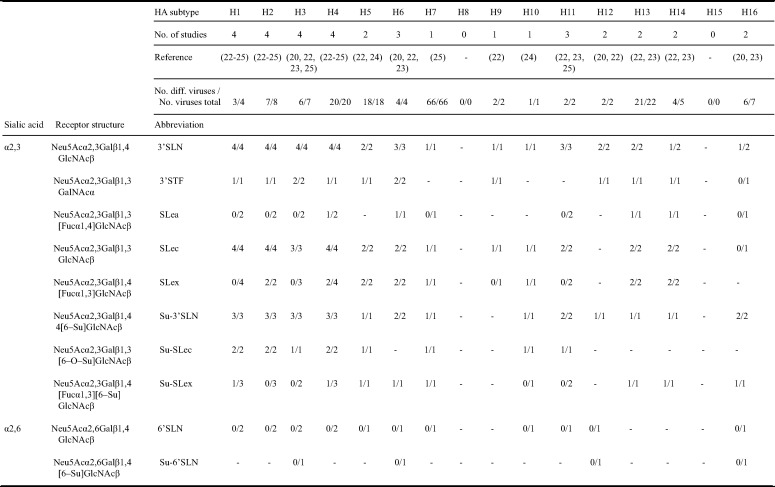
Summary of glycan attachment by low pathogenic avian influenza viruses based on the literature^*a*^

a This table summarizes previous studies on LPAIV-receptor attachment and lists glycan structures for which the attachment pattern to LPAIVs has been investigated in at least two glycan attachment studies. For each study, a positive score is listed for a receptor-subtype combination if >10% of the maximum attachment per subtype was observed (i.e., numerator). For each receptor-subtype combination, the number of studies is listed (i.e., denominator). –, receptor-subtype combination not investigated.

**TABLE 4:**
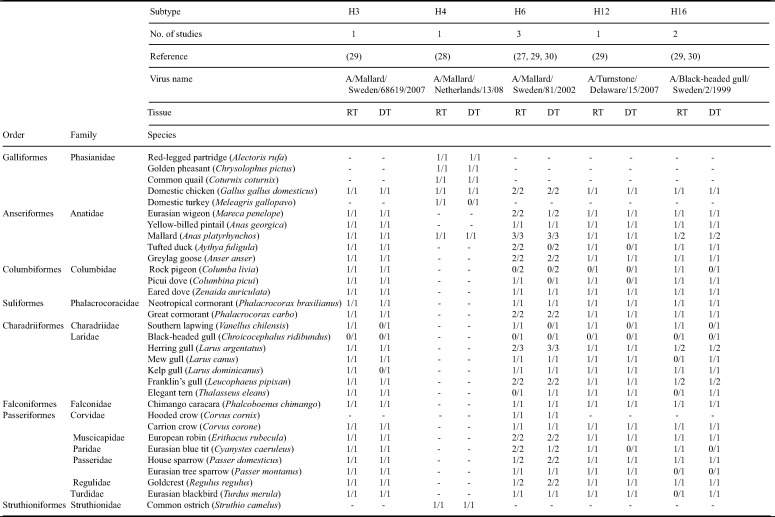
Summary of avian tissue attachment of low pathogenic avian influenza viruses based on the literature^*a*^

a This table summarizes previous studies on LPAIV-tissue attachment and lists all avian species investigated. For each study, a positive score is listed for a species-subtype combination if the virus attached to a tissue from at least one individual (i.e., numerator). For each species-subtype combination, the number of studies is listed (i.e., denominator). –, species-subtype combination not investigated; RT, respiratory tract; DT, digestive tract.

In this study, we investigated the research hypothesis that the level of attachment of IAVs H1 to H16 to mallard colon corresponds with their prevalence in mallards (Tables 1 and 2), with the most abundant attachment patterns for the common and intermediate subtypes and the least abundant attachment patterns for the rare subtypes. Next, we investigated the research hypothesis that the level of attachment of rare and intermediate subtypes is higher to colon of nonmallard duck species than to mallard colon. If attachment of these viruses is higher in nonmallard duck species, suggesting better adaptation to nonmallard duck species, then there may be a role for these nonmallard ducks as reservoirs for intermediate and/or rare IAV subtypes. To test these hypotheses, we performed attachment studies of Eurasian IAVs H1 to H16 to the intestinal tract of six duck species common in Eurasia, i.e., four dabbling duck species, namely, gadwall, Eurasian wigeon, mallard, and Eurasian teal, and two diving duck species, namely, common pochard and tufted duck. These duck species were chosen because of their abundance, preference for freshwater habitats, and migratory patterns in Eurasia. We considered virus attachment to the intestinal tract of importance, as this supports the fecal-oral transmission route as described for mallards. Furthermore, we performed attachment studies of IAVs H1 to H16 to the respiratory tract of the same six species of dabbling and diving ducks and to the respiratory and intestinal tract of specific-pathogen-free white leghorn chickens (Gallus gallus domesticus) to identify HA subtypes with an increased likelihood to switch between ducks and chickens. We considered virus attachment to the duck respiratory tract to be relevant for the ability of the virus to switch hosts from, e.g., duck (predominantly cloacal shedding) to, e.g., chicken (both tracheal and cloacal shedding) (31). In addition, to better understand the underlying mechanisms determining these virus attachment studies, we investigated the receptor attachment tropism of IAVs H1 to H16 to a glycan panel.

## RESULTS

### Prevalence of influenza A viruses and virus attachment patterns in colon of mallards.

**FIG 1:**
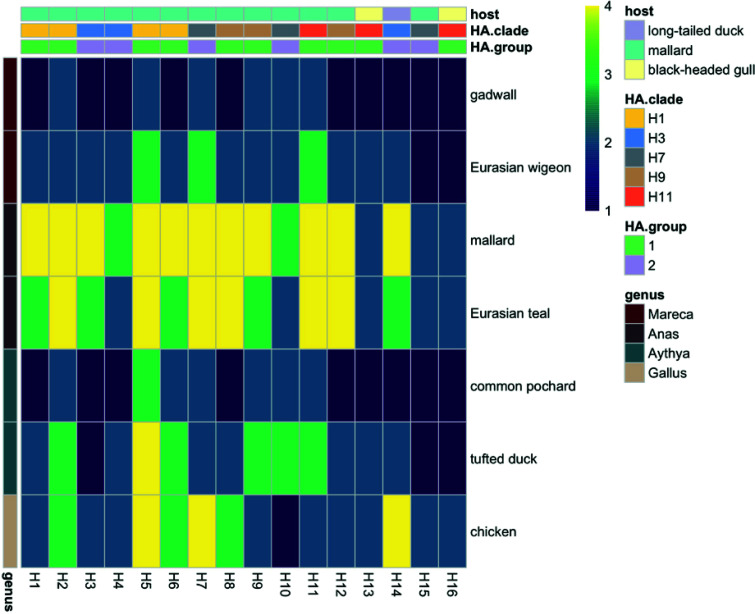
Attachment of low pathogenic avian influenza viruses of subtypes H1 to H16 to colon of six Eurasian duck species and chicken. The mean abundance of cells to which virus attached was scored as follows: 1, negative (no attachment); 2, scarce (<10% cells positive); 3, moderate (10 to 50% cells positive); 4, abundant (>50% cells positive).

All IAVs H1 to H16 attached to epithelial cells lining the colon of mallards (Fig. 1). Pairing of the H1 to H16 prevalence data (Table 1) to the level of virus attachment in colon of mallard (Fig. 1) was not statistically significant (Wilcoxon matched-pairs signed rank test, rs [nonparametric Spearman correlation coefficient]  = 0.3712, *P* = 0.0787). Nevertheless, subtypes considered “common” (H1 to H7 and H10) or “intermediate” (H8, H9, H11, and H12) (Table 1) attached most intensively to cells lining the intestinal tract of mallards, with H1 to H3, H5 to H9, H11, and H12 attaching to >50% of the cells and H4 and H10 attaching to 10 to 50% of the cells. Subtypes considered “rare” (H13 to H16) attached scarcely to cells lining the intestinal tract of mallards, with the exception of H14, which attached to >50% of the cells. Interindividual variation in virus attachment among mallards was small (median of absolute deviation [MAD] = 0 for H1 to H16) (Table 5).

**TABLE 5:**
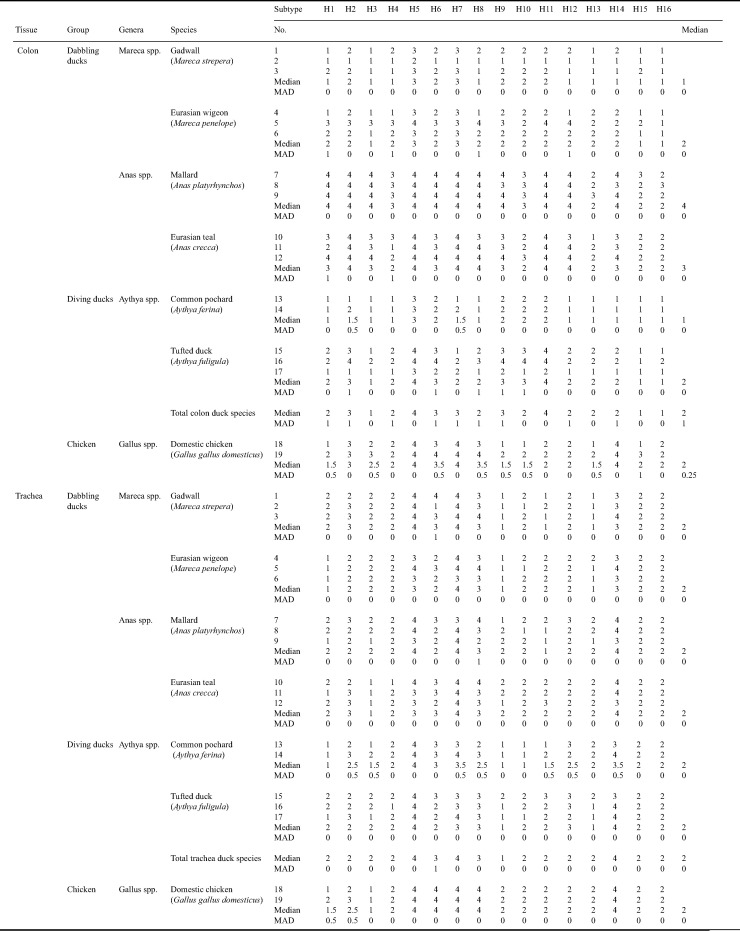
Attachment of low pathogenic avian influenza viruses of subtypes H1 to H16 to the colon and trachea of individual birds of six Eurasian duck species and chicken^*a*^

a The mean abundance of cells to which virus attached was investigated based on tissues from a total of 19 birds and was scored as follows: 1, negative (no attachment); 2, scarce (<10% cells positive); 3, moderate (10 to 50% cells positive); 4, abundant (>50% cells positive). MAD, median of absolute deviation.

### Attachment patterns of influenza A viruses in colon of mallards versus nonmallard ducks.

The level of overall H1 to H16 attachment in mallard colon was significantly different from that in colons of nonmallard duck species (Friedman test, FM [chi-square] 65.13, *P* < 0.0001). In general, IAV subtypes H1 to H16 attached significantly more intensively to epithelial cells lining the colon of mallard and Eurasian teal (*Anas* spp.) (Wilcoxon matched-pairs signed rank test, rs = 0.8386, *P* = 0.0002) than to cells lining the colon of gadwall and Eurasian wigeon (*Mareca* spp.). Of the diving duck species (*Aythya* spp.), fewer subtypes attached to cells lining the colon and generally less abundantly than to cells of mallard or Eurasian teal. Of the diving duck species, more subtypes attached to epithelial cells lining the colon of tufted duck than to cells of common pochard. Hence, mallard and Eurasian teal had similar abundant attachment to cells lining the colon, while Eurasian wigeon and tufted duck had similar scarce to moderate attachment to cells lining the colon (Wilcoxon matched-pairs signed rank test, rs = 0.6048, *P* = 0.0086). Gadwall and common pochard had the most scarce attachment to cells lining the colon (Wilcoxon matched-pairs signed rank test, rs = 0.8765, *P* = 0.0007). Subtypes H2, H5, H7, and H9 to H11 attached to colon of all dabbling and diving duck species, with the most abundant attachment of subtype H5. Subtype H3 did not attach to epithelial cells lining the colon of either diving duck species but did so to epithelial cells lining the colon of three of four dabbling duck species.

The level of attachment of rare and intermediate subtypes in mallard colon was significantly different from that in colons of nonmallard duck species (Friedman test, FM [chi-square] 35.02, *P* < 0.0001). Subtypes considered rare (H13 to H16) and intermediate (H8, H9, H11, H12) attached most abundantly in colon of mallard and Eurasian teal (Wilcoxon matched-pairs signed rank test, rs = 0.8944, *P* = 0.0179) and least abundantly in colon of gadwall and common pochard (same attachment scores), followed by Eurasian wigeon and tufted duck (Wilcoxon matched-pairs signed rank test, rs = 0.8909, *P* = 0.0119) (Fig. 1). Interindividual variation in virus attachment was lowest for mallard and gadwall (MAD = 0 for H1 to H16), followed by common pochard, Eurasian teal, Eurasian wigeon, and tufted duck (MAD = 0 for vast majority of H1 to H16 subtypes) (Table 5). Potential explanations for interindividual variation in virus attachment per HA subtype, in particular in colon of Eurasian wigeon and tufted duck, include differences in receptor expression due to genetic differences among individuals of the same species and tissue handling. Given that birds were approximately the same age and were fed the same food prior to tissue collection, these factors are less likely to explain interindividual differences.

### Attachment patterns of influenza A viruses in mallards and nonmallard ducks versus chickens.

**FIG 2:**
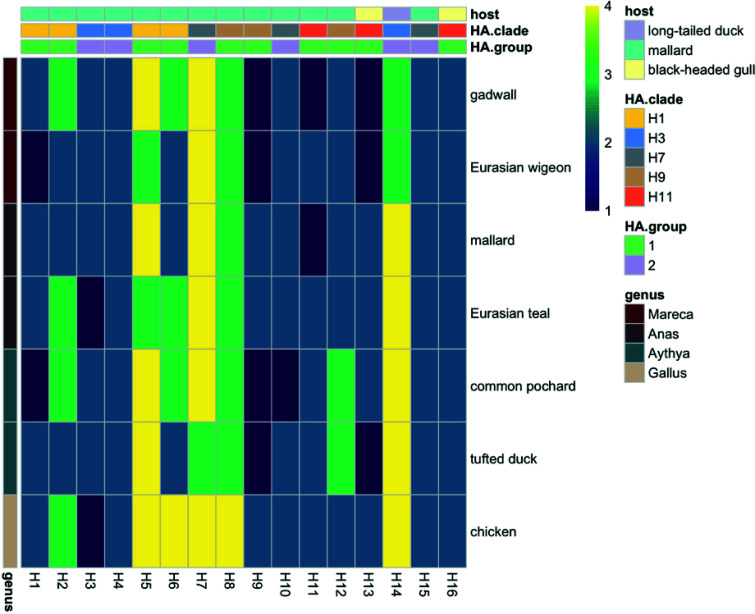
Attachment of low pathogenic avian influenza viruses of subtypes H1 to H16 to trachea of six Eurasian duck species and chicken. The mean abundance of cells to which virus attached was scored as follows: 1, negative (no attachment); 2, scarce (<10% cells positive); 3, moderate (10 to 50% cells positive); 4, abundant (>50% cells positive).

In chickens, the vast majority of IAV subtypes H1 to H16 attached to ciliated cells lining the trachea (Fig. 2) and to epithelial cells lining the colon (Fig. 1). Of these subtypes, H2, H5 to H8, and H14 attached most abundantly, with moderate to abundant attachment to both trachea and colon in chickens. This abundant attachment pattern of H2, H5 to H8, and H14 to trachea and colon fits with both tracheal and cloacal shedding. In *Anas* ducks, the same subtypes (H2, H5 to H8, and H14) attached moderately to abundantly to trachea (Fig. 2), while these and additional subtypes attached moderately to abundantly to colon (H1 to H12 and H14) (Fig. 1), in line with the predominant cloacal shedding in *Anas* ducks. In both ducks and chickens, the rare subtypes attached poorly to the trachea or colon, with the exception of H14.

In trachea of *Mareca* and *Aythya* ducks, the subtypes that attached most abundantly were H2, H5 to H8, H12, and H14, which was similar to the findings for trachea of *Anas* ducks and chickens, with the exception of H12. In contrast to *Anas* ducks, fewer subtypes and less abundant attachment was observed to colon than to trachea (Fig. 1). Of the subtypes that attached most abundantly to chicken trachea and colon (H2, H5 to H8, and H14), H5 and H7 were the two subtypes with the most abundant attachment also to cells lining the colon and trachea of dabbling and diving ducks investigated here (Fig. 1). Interindividual and interspecies variations in virus attachment patterns in the trachea were generally small for all duck species studied; each subtype attached to trachea of all individuals and species with similar intensities, either high or low (MAD = 0 for vast majority of H1 to H16 subtypes) (Table 5).

### Attachment patterns of influenza A viruses in ducks and chickens versus glycan attachment.

**FIG 3:**
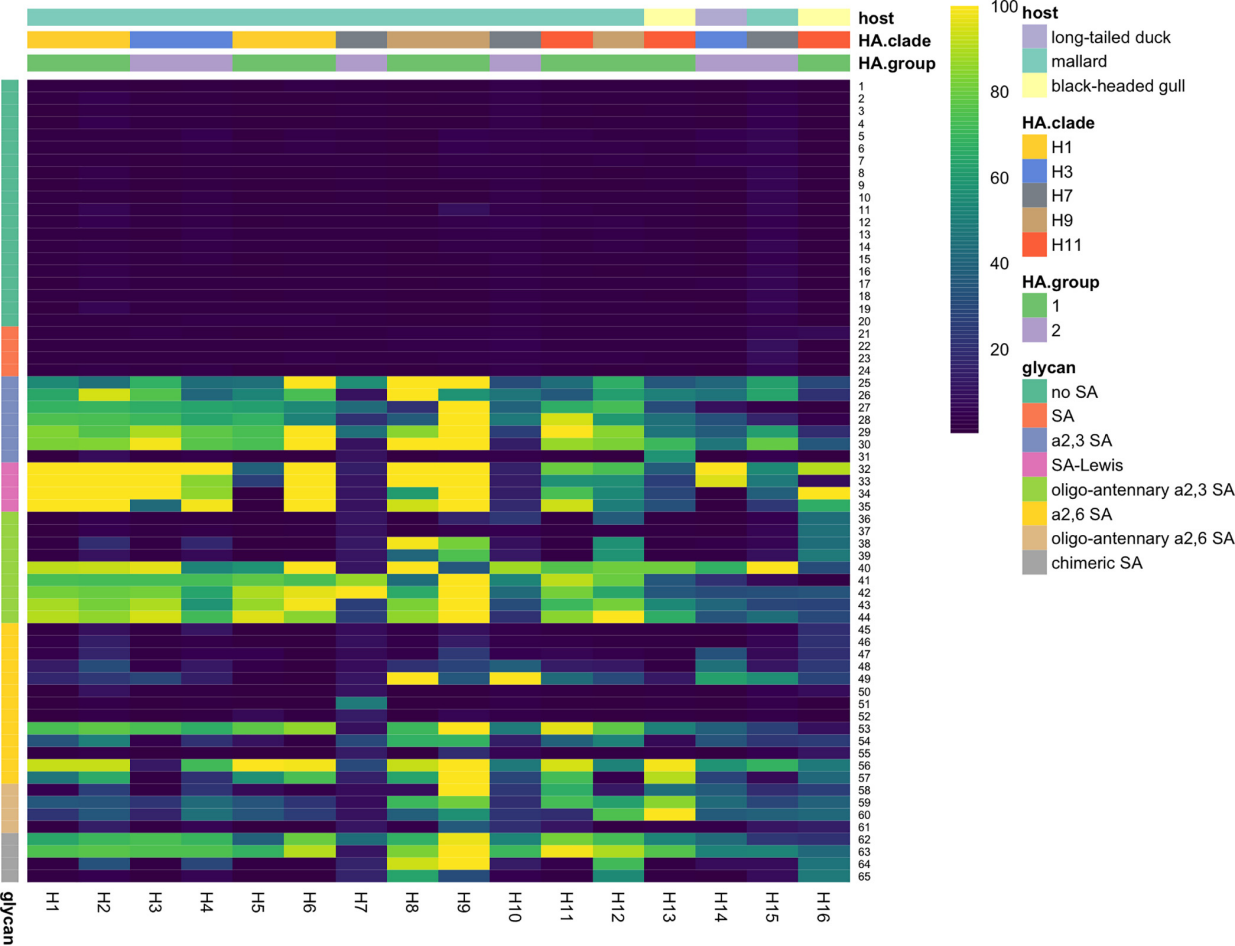
Attachment of low pathogenic avian influenza viruses of subtypes H1 to H16 to the glycan array. Each column represents one virus, and each row represents one glycan structure (Table 7). The colors indicate high (yellow) to low (dark blue) attachment based on the median signal from nine replicates for each virus and glycan structure combination, internally normalized toward the highest measured signal for each virus. The row annotation shows glycan type as shown in Table 7. Row numbers indicate glycan structures as shown in Table 7. The dendrogram was built based on Canberra distance and complete unweighted pair group method using average linkages (UPGMA) clustering of the attachment intensities. Column annotations show avian host species of virus isolation, HA clade, and HA group adopted from reference 69.

IAV subtypes H1 to H16 attached both to α2–3- and α2–6-linked SA structures, with generally more extensive attachment to α2–3-linked SA structures (structures 25 to 44) than to α2–6-linked SA structures (structures 45 to 61) (Fig. 3). The majority of the H1 to H16 viruses attached to the 41 sialylated glycans (structures 25 to 65), while no viruses attached to the 20 nonsialylated glycans (structures 1 to 20) or to the four plain SAs (structures 21 to 24). Most of the HA subtypes showed high attachment to fucosylated sialylated structures (structures 32 to 35), yet the effect of fucosylation depended on subtype and glycan structure. Also, most of the HA subtypes showed high attachment to sulfated sialylated structures, but attachment differed per HA subtype, glycan, and position of the sulfate group.

The common and intermediate subtypes in mallards (i.e., H1 to H12) showed high attachment to 3′SLN biantennary N-glycan (α2–3 SA oligoantennary N-glycan structure; structure 41) and the majority of H1 to H12 subtypes showed high attachment to 3′SLN and 3′STF (structures 27 and 28), in contrast to rare subtypes (i.e., H13 to H16), which showed low attachment to these structures. Furthermore, the majority of H1 to H12 subtypes that attached to 3′SLN and 3′STF showed similar or slightly more attachment to the fucosylated (structures 32 nd 33) or sulfated (structures 29 and 30) structure analogues, while rare subtypes showed more attachment to sulfated (H13 to H16) and in particular fucosylated (H14 to H16) structures than to their nonsulfated or nonfucosylated structure analogues. HA subtypes considered intermediate (i.e., H8, H9, H11, and H12) subtypes in mallards and belonging to genetic clade 9 (i.e., H8, H9, and H12) and the rare subtype H16 in mallard showed high attachment to α2–8-linked Neu5Ac oligomers (structures 38, 39, 64, and 65). The abundant attachment of the rare H14 subtype to the epithelial cells lining the colon of mallards was associated with high attachment of H14 to SLe^a^ (structure 33), compared to H13, H15, and H16 viruses, which showed less attachment to this structure. A few rare subtypes were the only subtypes that attached to some of the glycan structures, i.e., H13 to GalNAcβ1,4[Neu5Acα2–3]Galβ1,4Glc (structure 31; 58%) and H16 to GalNAcβ4[Neu5Acα2–8Neu5Acα2–3]Galβ1,4Glc (structure 37; 45%).

The subtypes that attached to epithelial cells lining the colon of all dabbling and diving duck species (i.e., H2, H5, H7, and H9 to H11) were as a group not associated with high attachment to a single, specific glycan structure. Similarly, the subtypes that attached most abundantly to ciliated cells lining the trachea and epithelial cells lining the colon in chickens (i.e., H2, H5 to H8, H14) were as a group not associated with high attachment to a single, specific glycan structure. Yet, of those subtypes, H5 and H7 showed less attachment to fucosylated structures SLe^x^ and SLe^a^ (structures 32 and 33) than to the nonfucosylated structure analogues (structures 27 and 28). In addition, H7 showed a distinct glycan attachment: while the majority of the investigated viruses attached to the 40 glycans terminating with Neu5Ac, only the H7 virus attached to the single glycan terminating with Neu5Gc (Neu5Gc-LN) (structure 51; 49%). The H8 isolate showed more attachment to sulfated α2–3-linked SA and α2–3 Lewis structures (structures 29, 30, 34, and 35) than to nonsulfated structure analogues (structures 27 and 32), while both H5 and H14 showed no attachment to sulfated α2–3 Lewis structures (0% and 1% for H5 and H14; structures 34 and 35) and moderate to high attachment to the nonsulfated structure analogue (38% and 100% for H5 and H14; structure 32).

## DISCUSSION

Here we investigated the host range of avian IAVs H1 to H16 based on virus attachment patterns to the respiratory and intestinal tract of six Eurasian duck species and chicken. In addition, we investigated the attachment of the same viruses to a panel of 65 synthetic glycan structures. First, the “common” (i.e., H1 to H7 and H10) and “intermediate” (i.e., H8, H9, H11, and H12) subtypes in mallards had moderate to abundant attachment to mallard colon. Yet, as not all “rare” subtypes (i.e., H13 to H16) had scarce attachment to mallard colon, the null hypothesis (i.e., the level of attachment of H1 to H16 IAVs to mallard colon is independent of their prevalence in mallards) could not be falsified. Thus, the level of attachment of H1 to H16 to mallard colon did not correspond completely with mallard surveillance reports. Second, the most abundant attachment of rare and intermediate subtypes to colon was observed in mallards in comparison to the other investigated species, therefore falsifying the null hypothesis (i.e., the levels of attachment of rare and intermediate subtypes to mallard and nonmallard duck colon are the same). Yet, the colon attachment patterns in ducks do not support the research hypothesis (i.e., the level of attachment of rare and intermediate subtypes is higher to colon of nonmallard duck species than to mallard colon) and suggests that mallards may be the reservoir to some intermediate subtypes and/or that intermediate and rare subtypes have a reservoir host other than the species tested here (e.g., H13 and H16 in gulls).

### Extensive virus attachment to colon of *Anas* ducks versus colon of other duck genera.

The subtypes that are common and intermediate in mallards generally attached more abundantly to colon of *Anas* species than to colon of *Mareca* or *Aythya* species, while the subtypes that are rare in mallards, with the exception of H14, attached equally poorly to the colon of *Anas* species and that of *Mareca* and *Aythya* species. The majority of viruses investigated here have been isolated from mallards. So far there is no evidence that LPAIVs of the same HA subtype but isolated from different duck species differ in their receptor binding patterns (e.g., H7 [25] and H4 [23]), in contrast to H4 LPAIVs isolated from ducks and gulls, which are taxonomically less closely related (23). HA amino acid position 222 has been suggested to be an important determinant of the receptor specificity (e.g., H5 [32]), and substitutions have been observed when viruses are transmitted from ducks to gulls or shorebirds (23). Thus, given the lack of evidence of receptor-binding adaptation among duck virus isolates based on receptor attachment studies done previously, and the low attachment of mallard isolate H15 virus to mallard tissue in our study, we do not expect a strong bias effect toward mallards due to the fact that 13 of 16 viruses were isolated from mallards. Actually, the predominantly scarce attachment of the majority of HA subtypes to colon of *Mareca* and *Aythya* species mirrors the low diversity of HA subtypes detected in these species in nature (Table 2). Therefore, the low virus prevalence and diversity detected in Eurasian wigeon and tufted duck, followed by gadwall and common pochard sampled as part of surveillance programs, may be due to species-specific susceptibility to infection of the digestive tract with a smaller range of HA subtypes in addition to the oft-cited lower sampling efforts (6, 33, 34). Given the difference in virus attachment to colon of *Anas* species versus *Mareca* and *Aythya* species, and potentially host range, the role of different ducks in IAV epidemiology may be less shaped by dabbling versus diving ducks than by genus *Anas* versus other duck genera.

### No evidence for *Anas*, *Mareca*, or *Aythya* ducks to be reservoir hosts of rare subtypes.

Attachment patterns of the rare subtypes to colon of the investigated duck species suggested no or a minimal role for these species as hosts for H13, H15, and H16. The absent to scarce attachment of H13 and H16 corresponds with the low detection of these subtypes in ducks in general. Gulls are the primary source for H13 and H16 viruses (9), in which they can cause annual outbreaks on colony breeding sites. The poor attachment of the investigated H16 isolate to colon from the duck species was in line with earlier findings with another H16 isolate (29, 30). Our findings support the H13 and H16 Laridae versus Anseriformes host discrimination reported from field surveillance. In contrast to H13 and H16, the primary source of H15 viruses has not yet been identified. Surprisingly, as H15 attached only scarcely to colon of mallard and Eurasian teal, the few reported H15 viruses in Eurasia were detected in these species (35, 36). An explanation for the rare H15 detection may be that these birds carried the H15 virus without attachment and infection and had obtained the virus from a neglected, undersampled species. Alternatively, H15 virus attached and replicated in a part of the intestinal tract other than the colon or in another organ, or in the colon, as scarce attachment does not exclude the possibility of infection. Most remarkable of the rare subtypes was H14, which attached moderately to abundantly to colon of Eurasian teal and mallard, suggesting that these species may be susceptible to infection with this subtype. The H14 subtype has been isolated a few times, mainly from blue-winged teals (Anas discors) in South America but only once from wild birds in Eurasia (37–40), which may have been mingling with the unsampled, unidentified reservoir host. H14 IAV may not recently have been successful in circulating in Eurasia due to competition among HA subtypes with respect to replication efficiency, transmission efficiency (including environmental survival), immunogenicity (affecting reinfection of the same host) (41), evasion of the host immune system (42, 43) (in particular with common subtypes H3 and H4 belonging to the same clade [41, 44]), and genetic reassortment with, e.g., different NA subtypes and internal genes affecting replication and transmission. Alternatively, H14 IAV may circulate in Eurasia but remain undetected because the reservoir hosts are not included in surveillance programs.

### Subtypes H2, H5 to H8, and H14 are more likely to switch between wild ducks and chickens.

The virus attachment patterns of subtypes H1 to H16 in the trachea of chickens were the same as those in the trachea of the *Anas*, *Mareca*, and to large extent *Aythya* ducks, with moderate to abundant attachment of H2, H5 to H8, and H14 (Fig. 2). Therefore, we hypothesize that viruses that attach moderately to abundantly to the epithelial cells lining the trachea of ducks may be more likely to attach to, and potentially infect, the trachea of chickens (45), which is in agreement with high LPAIV H2 and H5 to H8 prevalence in poultry in a long-term surveillance study (45). Although H9 appears to be common in poultry, this H9 representative showed low virus attachment to the chicken tissues in this study. Yet, besides the HA protein, which plays an important role in virus attachment, fusion, environmental persistence, and the influenza-specific immune response, the infection and transmission of LPAIVs are shaped by the remaining genomic segments and their products and the interaction with the host cellular machinery. Common H3 and H4 subtypes in mallard attached abundantly to mallard colon but scarcely to mallard trachea and therefore may be less likely to switch between mallards and chickens. Indeed, subtypes H3 and H4 have been rarely detected in poultry (15, 45). However, experimental infections have suggested that chickens can become infected with H3 and H4 IAV (46). Virus attachment patterns in the chicken trachea were the same as those in the chicken colon, which fits with both tracheal and cloacal shedding. In contrast to chickens, virus attachment patterns to trachea in ducks were intense and consistent, with little interspecies and interindividual variation (similar to observations of Eriksson et al. [29]), compared to the large interspecies and interindividual variation seen in colon.

The abundant attachment of H2, H5 to H8, and H14 to *Anas* duck colon suggested an important role for *Anas* ducks as the host of the IAV that may infect chickens and fits with the predominant cloacal IAV shedding of *Anas* ducks. In addition, H5 showed the most abundant attachment in colon (and trachea) in all duck species, which corresponded with the broad species distribution of H5 described in literature and based on IAV sequence databases (Tables 1 and 2) (37, 47). Thus, the frequent detection of H5 within wild bird surveillance programs might be due not only to diagnostics targeting H5 and H7 IAV but also to the possibility that LPAIV H5 has a broader host range than, e.g., H3 and H4 subtypes, based on abundant virus attachment in this study.

### Limited evidence for shared glycan attachment patterns of common, intermediate, or rare subtypes.

The distribution of α2–3- and α2–6-linked SA in birds seems to be tissue and species dependent. For instance, in mallards, both α2–3- and α2–6-linked SA have been detected on the surface of the cells lining the respiratory tract (28, 48–50), while α2–3-linked SA was most often detected on cells lining the intestinal tract (28, 48, 49). More recently, different proportions of α2–3- and α2–6-linked SA were detected in the colon of mallard (α2–3 ≈ α2–6), Eurasian wigeon (α2–3 < α2–6), and tufted duck (α2–3 < α2–6) (29). This may explain why in our study some HA subtypes (e.g., H3) attached abundantly to mallard colon but not or scarcely to colon of Eurasian wigeon or tufted duck. Here, the majority of common and intermediate HA subtypes (H1 to H12) showed high attachment to the following α2–3-linked SA structures: 3′SLN biantennary N-glycan, 3′SLN, and 3′STF. In contrast, rare subtypes showed very low attachment to the 3'SLN biantennary N-glycan, 3′SLN, and/or 3′STF. This suggests that LPAIV gull isolates (here H13 and H16) are not unique in their low attachment to 3′SLN and 3′STF but that other nongull, wild bird isolates (here H14 and H15) have the same limited attachment to 3′SLN and 3′STF as LPAIV gull isolates. The data presented here suggest that tropism to fucosylated structures is more general among IAV subtypes, as the majority of common and intermediate duck-originating HA subtypes showed high attachment to fucosylated structures, including SLe^x^ and Su-SLe^x^. The attachment of LPAIVs to fucosylated glycan structures was previously reported to be specific for chicken or gull IAVs (22, 23, 25). Despite this extensive attachment to fucosylated structures, the subtypes H5, H7, and H14 seem to attach more to nonfucosylated and nonsulfated structures than to their fucosylated and sulfated structure analogues. Interestingly, these HA subtypes showed here the most extensive attachment in duck trachea (H5, H7, H14) and in duck colon (H5), while limited data supported the absence of fucosylated glycans in epithelial cells lining the intestinal tract of ducks (24, 32, 51, 52). The majority of H1 to H12 subtypes and H14 showed high attachment to SLe^a^ (α2–3-fucosylated SA, Lewis). Given the unexpected abundant attachment of H14 to colon and trachea of ducks and chickens, and high attachment of H14 to SLe^a^, we hypothesize that SLe^a^ may have a role in H14 attachment to epithelial cells lining the intestinal tract of *Anas* ducks, in line with the abundant attachment of subtypes H1 to H12. Lastly, the studied H7 isolate was the only subtype that attached to 6′Neu5Gc-LN. Attachment to 3′Neu5Gc-LN has been described for H3 duck viruses (22) and recombinant H5 IAV (53). However, attachment of wild-type IAV to 6′Neu5Gc-LN has not been described (53). The Neu5Gc molecule is reported not to be present in birds or humans, while this molecule (3′ and/or 6′ linked) is commonly expressed in trachea of horses and pigs (53). Attachment to 3′Neu5Gc(-LN) has been observed for equine H7N7 viruses that caused a severe outbreak in horses (25, 53), yet the structure 3′Neu5Gc-LN was not incorporated in our glycan panel and could therefore not be tested.

The observed virus attachment patterns in this study are based on a single representative LPAIV per HA subtype. Prior studies on avian tissue attachment of LPAIVs were performed based on a single LPAIV per HA subtype (i.e., H3, H4, H6, H12, or H16) (Table 4) (27–30); therefore, evidence is lacking for potential differences in avian tissue attachment patterns among LPAIVs of the same HA subtype. However, prior studies on receptor attachment with multiple representatives per LPAIV subtype (e.g., *n* = 20 for H4, *n* = 18 for H5, *n* = 66 for H7, *n* = 21 for H13) (20, 22–25) provide some evidence that some LPAIV HA subtypes (i.e., H5 and H7) show more similar attachment patterns within the same subtype than others (i.e., H4 [duck versus gull isolates] and H13 [gull isolates, group 1 and group 2]). Thus, by using a single representative per HA subtype, we might have missed within-subtype variation in receptor attachment patterns for some subtypes, e.g., H4 and H13. Furthermore, prior to attachment analyses, viruses were passaged in embryonated chicken eggs. The passage of IAVs in embryonated chicken eggs can indeed result in genetic changes that may affect virus function, including attachment (54, 55). The latter has not been demonstrated for LPAIVs, yet egg adaptation related to receptor binding has been shown for human influenza A (H3N2) vaccine viruses (56).

In conclusion, the observed virus attachment patterns partially explained the reported field surveillance LPAIV subtype distribution in ducks. Indeed, virus attachment was the most intense and widespread in colon of the mallard and the Eurasian teal, supporting the importance of *Anas* ducks as hosts of AIVs, including those infecting chicken, and supporting the fecal-oral transmission route. The reported glycan attachment profile did not explain the virus attachment patterns to colon and/or trachea but did provide new information on receptor binding specificity of LPAIVs, including H7 tropism for 6′Neu5Gc and significant attachment of LPAIVs to fucosylated glycan structures. The expression of glycan structures in the avian digestive tract is largely unknown yet may vary due to changes in diet (57), infections (58), and possibly age (59), as previously shown for nonbird species. Future studies should aim to identify and validate glycan receptors (including α2–8-linked Neu5Ac oligomers) on the intestinal mucosa of birds, as well as replication in avian cell lines, to better understand the interplay between IAVs and their receptors and hosts. The knowledge gained in this study will be of value to further investigate the IAV receptor binding specificity and to optimize wild bird surveillance programs.

## MATERIALS AND METHODS

### Ethics.

The tissues were obtained from a tissue bank at the Erasmus Medical Center (MC) and had been used as negative controls in an infection experiment (60) that was approved by the Dutch Animal Ethical Committee.

### Virus preparation.

The IAVs used in this study (Table 6) were obtained from fecal swabs of wild birds during ongoing influenza virus surveillance and subsequently passaged 2 or 3 times in embryonated chicken eggs. The IAVs of the HA subtypes H1 to H13 and H15 and H16 were obtained from the Ottenby Bird Observatory in southeast Sweden. Of these, the H13N8 and H16N3 viruses were isolated from black-headed gulls (Chroicocephalus ridibundus), and the other viruses were from mallards (Table 6). Influenza A virus A/long-tailed duck/Wisconsin/10OS3912/2010 (H14N6) was kindly provided through the Animal Influenza Ecology and Epidemiology Research Program (Ohio State University, USA), as this HA subtype had not been detected recently in Sweden or elsewhere in Europe. These viruses were selected because they represent all HA subtypes isolated from birds (H1 to H16), originate from the same sampling area (with the exception of H14), were recently isolated (from 2003 onwards), and were of common HA-NA combinations (14). Viruses were prepared and inactivated using the method described by van Riel et al. (61). The HA titer of the virus isolate was determined using a hemagglutination assay.

**TABLE 6:** Low pathogenic avian influenza viruses used in this study to investigate tissue and receptor binding patterns^*a*^

Virus subtype	Virus name	Egg passage	GenBank accession no.	Amino acid at indicated position near the receptor binding site of the HA protein											
				98	138	190	215	222	223	224	225	226	227	228	229
H1N1	A/mallard/Sweden/104803/2009	2	JX566076	Y	A	E	P	K	V	N	G	Q	A	G	R
H2N3	A/mallard/Sweden/105050/2009	2	KC342630	Y	A	E	P	K	V	N	G	Q	G	G	R
H3N8	A/mallard/Sweden/101487/2009	3	KT725399	Y	A	E	P	W	V	R	G	Q	S	G	R
H4N6	A/mallard/Sweden/80148/2008	2	CY165570	Y	A	E	P	W	V	R	G	Q	S	G	R
H5N2	A/mallard/Sweden/74/2003	2	CY076929	Y	A	E	P	K	V	N	G	Q	S	G	R
H6N2	A/mallard/Sweden/99825/2009	NA	JX565996	Y	A	E	P	A	V	N	G	Q	R	G	R
H7N7	A/mallard/Sweden/5944/2005	2	CY184584	Y	A	E	P	Q	V	N	G	Q	S	G	R
H8N4	A/mallard/Sweden/58256/2006	3	CY183531	Y	A	E	P	L	V	R	G	Q	Q	G	R
H9N2	A/mallard/Sweden/67860/2007	NA	CY184149	Y	A	E	P	L	V	R	G	Q	Q	G	R
H10N1	A/mallard/Sweden/102087/2009	NA	CY183839	Y	A	E	P	Q	V	N	G	Q	S	G	R
H11N9	A/mallard/Sweden/102103/2009	NA	CY184189	Y	A	E	P	K	V	N	G	Q	A	G	R
H12N5	A/lure duck/Sweden/100127/2009	2	JX566037	Y	A	E	P	L	V	R	G	Q	Q	G	R
H13N8	A/black-headed gull/Sweden/55215/2006	3	KR087597	Y	A	E	L	G	Y	N	G	Q	K	S	W
H14N6	A/long-tailed duck/Wisconsin/10OS3912/2010	3	JN696314	Y	A	E	P	R	V	R	N	Q	S	G	R
H15N5	A/mallard/Sweden/139647/2012	2	MF147992	Y	A	E	P	K	V	N	G	Q	A	G	R
H16N3	A/black-headed gull/Sweden/87533/2009	2	CY184496	F	S	T	L	G	Y	N	G	Q	K	S	W

a The low pathogenic avian influenza viruses shown here were used to investigate attachment patterns to colon and trachea of six duck species and to glycan structures. Lure duck is mallard. The egg passage number indicates the number of passages of the virus in embryonated chicken eggs before the virus was used in this study. NA, not available.

### Tissue preparation.

Archival samples of colon and trachea of gadwall, Eurasian wigeon, mallard, Eurasian teal, common pochard, tufted duck, and white leghorn chickens were obtained from the Department of Viroscience, Erasmus MC. Tissue samples had been fixed in 10% neutral buffered formalin for 48 h, embedded in paraffin, and stored at room temperature in paraffin blocks. Two to three individuals per species were analyzed (Table 5). Ducks were 8 to 11 months of age, and chickens were 4 to 6 weeks of age. All tissues selected were from individuals without histological lesions and that tested negative for IAV and IAV-specific antibodies at postmortem examination.

### Virus histochemistry.

To investigate virus attachment patterns to tissue sections, histochemical analysis as described previously was performed (61). A positive result was visible by light microscopy as granular to diffuse red staining on the apical surface of epithelial cells in the colon or trachea. Mean abundance of cells to which virus attached was scored as follows: −, negative (no attachment); ±, scarce (<10% cells positive); +, moderate (10 to 50% cells positive); ++, abundant (>50% cells positive). For each bird and tissue, 16 transverse sections were incubated with fluorescein isothiocyanate (FITC)-labeled IAV and an omission control was included to check for unspecific staining. Archival paraffin-embedded colon tissue from a mallard with FITC-labeled H3 LPAIV was included as a positive control.

### Glycan array.

To investigate virus attachment patterns to glycan structures, an array comprising 65 different synthetic glycans per well was used (Table 7). This array contained nonsialylated glycans (structures 1 to 10), sulfated nonsialylated glycans (structures 11 to 20), SA (structures 21 to 24), α2–3-linked SA (structures 25 to 31), fucosylated α2–3-linked SA (SA-Lewis) (structures 32 to 35), oligoNeu5Ac (structures 36 to 40 and 62 to 65), α2–3-linked SA oligoantennary N-glycans (structures 41 to 44), α2–6-linked SA (structures 45 to 57), and α2–6-linked SA oligoantennary N-glycans (structures 58 to 61). Structures 29, 30, 34, 35, 53, and 54 were sulfated. Glycan array analysis was performed by following described protocols (20, 62). The fluorescence signal was measured by a ScanArray GX microarray scanner (PerkinElmer) and analyzed using ProScanArray Express version 4.0 (PerkinElmer) and RStudio 1.0.136 (RStudio Core Team [2016], Vienna, Austria). The median signal was calculated from nine replicates for each virus and glycan structure combination and then internally normalized toward the highest measured signal for each virus. Values of <3% were regarded as baseline (63). Heat maps of obtained scores were constructed using the pheatmap R package (64–68).

**TABLE 7:**
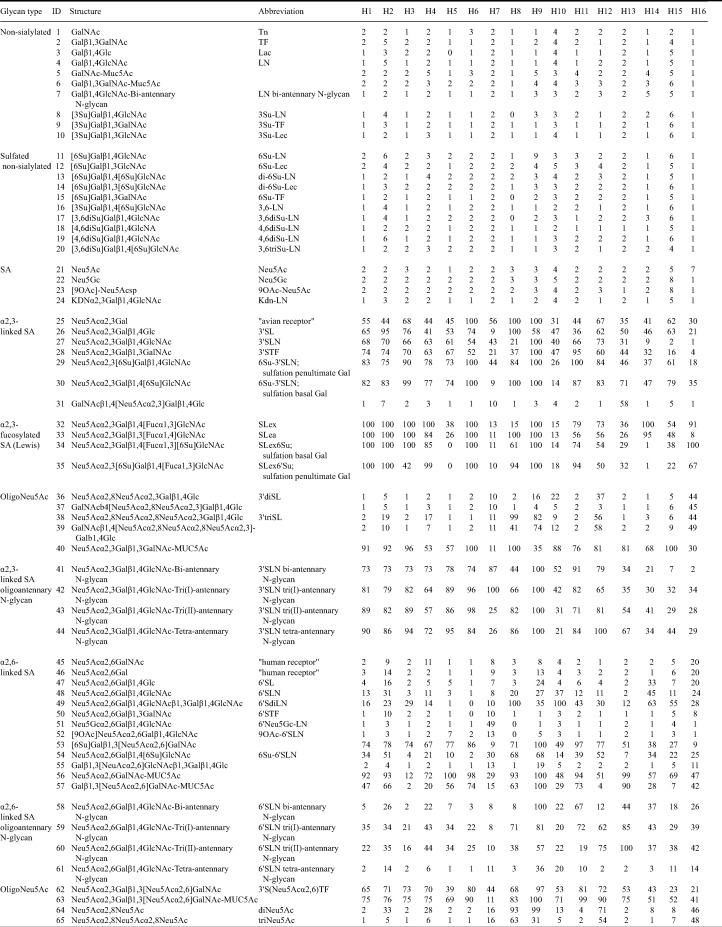
Attachment of low pathogenic avian influenza viruses of subtypes H1 to H16 to glycan structures^*a*^

a Attachment patterns are shown based on the median signal from nine replicates for each virus (*n* = 16) and glycan structure (*n* = 65) combination, internally normalized toward the highest measured signal for each virus. The type column describes the glycan type. The ID column denotes the glycan number indicated in Fig. 3. The structure column shows the glycan structure, and the abbreviation column shows the abbreviations used if applicable. SA, sialic acid; Su, sulfate group; Fuc, fucosylated.

### Statistical analyses.

The Wilcoxon matched-pairs signed rank test was used to compare IAV subtype prevalences in mallards and virus attachment patterns in mallard colon. The Friedman test was used to detect significant differences between virus attachment patterns in the different duck species. Analyses were performed using GraphPad Prism 8.0. The median of absolute deviation was calculated using Microsoft Excel v16.16.27.

### Data availability.

Sequences are available in GenBank under accession numbers JX566076, KC342630, KT725399, CY165570, CY076929, JX565996, CY184584, CY183531, CY184149, CY183839, CY184189, JX566037, KR087597, JN696314, MF147992, and CY184496.
